# Egg consumption and risk of all-cause and cause-specific mortality in an Italian adult population

**DOI:** 10.1007/s00394-021-02536-w

**Published:** 2021-03-24

**Authors:** Emilia Ruggiero, Augusto Di Castelnuovo, Simona Costanzo, Mariarosaria Persichillo, Amalia De Curtis, Chiara Cerletti, Maria Benedetta Donati, Giovanni de Gaetano, Licia Iacoviello, Marialaura Bonaccio, the Moli-sani Study Investigators, Licia Iacoviello, Giovanni de Gaetano, Maria Benedetta Donati, Marialaura Bonaccio, Americo Bonanni, Chiara Cerletti, Simona Costanzo, Amalia De Curtis, Augusto Di Castelnuovo, Francesco Gianfagna, Mariarosaria Persichillo, Teresa Di Prospero, Jos Vermylen, Ignacio De Paula Carrasco, Antonio Spagnuolo, Deodato Assanelli, Vincenzo Centritto, Simona Costanzo, Marco Olivieri, Teresa Panzera, Augusto Di Castelnuovo, Marialaura Bonaccio, Simona Costanzo, Simona Esposito, Alessandro Gialluisi, Francesco Gianfagna, Emilia Ruggiero, Amalia De Curtis, Sara Magnacca, Benedetta Izzi, Annalisa Marotta, Fabrizia Noro, Roberta Parisi, Alfonsina Tirozzi, Mariarosaria Persichillo, Francesca Bracone, Francesca De Lucia, Cristiana Mignogna, Teresa Panzera, Livia Rago, Americo Bonanni

**Affiliations:** 1 Department of Epidemiology and Prevention, IRCCS NEUROMED, Via dell’Elettronica, 86077 Pozzilli, IS Italy; 2 Mediterranea Cardiocentro, Napoli, Italy; 3 Research Centre in Epidemiology and Preventive Medicine (EPIMED), Department of Medicine and Surgery, University of Insubria, Varese-Como, Italy

**Keywords:** Eggs, Dietary cholesterol, Mortality risk, Mediterranean diet

## Abstract

**Purpose:**

Dietary guidelines recommend to limit egg consumption to 4 servings per week but the relation between egg intake and health outcomes is still controversial. To evaluate the association of egg consumption and mortality risk in Italian adults and to investigate nutritional factors and serum lipids as potentially explaining such associations.

**Methods:**

Longitudinal analysis on 20,562 men and women aged ≥ 35y, free from cardiovascular disease (CVD) and cancer belonging to the Moli-sani Study cohort (enrolled 2005–2010) followed up for a median of 8.2 years.

**Results:**

In multivariable-adjusted analysis as compared to low intake (> 0 ≤ 1 egg/week), eating > 4 eggs/week led to an increased risk of all-cause (Hazard ratio [HR] = 1.50; 95%CI 1.13–1.99), CVD (HR = 1.75; 1.07–2.87) and cancer mortality (HR = 1.52; 0.99–2.33). Similarly, an intake of 2–4 eggs/week was associated with higher all-cause (HR = 1.22; 1.01–1.46) and CVD mortality risk (HR = 1.43; 1.03–1.97). An increase of 1 egg per week was associated with higher mortality risk among high-risk individuals, such as those with hypertension and hyperlipidaemia. Dietary cholesterol explained about 43.0% and 39.3% (*p* values < 0.0001) of the association of eggs with all-cause and CVD mortality, respectively, while serum lipids (*e.g.,* total cholesterol) accounted for a small proportion of egg-mortality relation.

**Conclusions:**

Among Italian adults, high egg consumption leads to an increased risk of all-cause and CVD mortality, with the risk being evident even at the recommended intake of 2–4 eggs per week. A substantial part of this association was likely due to the egg contribution to dietary cholesterol. Our findings suggest limiting the consumption of eggs in the diet and these results should be considered in the development of dietary guidelines and updates.

**Supplementary Information:**

The online version contains supplementary material available at 10.1007/s00394-021-02536-w.

## Introduction

Eggs are a common food in every diet and are used in many basic and formulated preparations. Eggs are affordable and nutrient-dense food items, containing high-quality protein with low levels of saturated fatty acids, and are rich in several micronutrients including vitamins and minerals, some of which (vitamin E, carotenoids) are reported to have antioxidant properties [1].

On the other side, eggs are also major sources of dietary cholesterol, a potential risk factor for cardiovascular health [2]. Dietary guidelines for egg consumption have changed several times over the past few decades, and vary among health agencies [3, 4]. In 2010, the Dietary Guidelines for Americans recommended cholesterol intake to be limited to no more than 300 mg per day [5], but the 2015–2020 Dietary Guidelines Advisory Committee did not bring forward this recommendation, due to the lack of evidence of an appreciable relationship between consumption of dietary cholesterol and serum cholesterol, a decision consistent with the conclusions of the American Heart Association/American College of Cardiology report [6].

Based on their high cholesterol content, the Mediterranean Diet Foundation recommends to consume up to 4 eggs per week, as a healthy alternative to fish or meat [7], and the same amount (2–4 eggs per week) was indicated in the latest Italian dietary guidelines [8]. In the Eatwell Guide issued by the National Health Service in the UK there is no recommended limit on how many eggs people should eat [9].

Epidemiological studies analysing the association of eggs with health outcomes have provided controversial results [10–12]. Several meta-analyses and an umbrella review of observational studies failed to report significant associations between egg intake and cardiovascular outcomes, even highlighting a downward trend association with stroke risk [13–16].

Data on the association of egg intake with mortality risk is limited and controversial [17–19], and the scientific evidence for the recommendations on dietary cholesterol and eggs is inconsistent and lacking in Mediterranean countries where the association between eggs and health has been seldom explored [10, 20, 21].

In light of this, our study first aimed to longitudinally evaluate the association of egg consumption with all-cause and cause-specific mortality in a large Italian population of adult men and women; second, to investigate whether dietary cholesterol and other nutritional factors may account for the association between egg intake and mortality risk.

## Materials and methods

### Study population

Data are from the Moli-sani Study cohort established in 2005–2010 in the Molise region, a Mediterranean area in Italy, that recruited 24,325 men and women aged ≥ 35 years [22].

For the purpose of the present analyses, we excluded subjects with a history of cardiovascular disease (CVD; *n* = 1537) or missing data on CVD (*n* = 360), with a history of cancer (*n* = 777) or missing data on cancer (*n* = 89), those individuals with missing data for egg intake (*n* = 100), those reporting implausible energy (< 800 kcal/d in men and < 500 kcal/d in women or > 4000 kcal/d in men and > 3500 kcal/d in women; *n* = 771) or egg intakes (≥ 14 eggs/week; *n* = 2), dietary or medical questionnaires judged as unreliable by interviewers (*n* = 955 and *n* = 235, respectively), subjects lost to follow-up (*n* = 23) and missing information on cause-specific death (*n* = 45).

We finally analysed 20,562 individuals (84.5% of the study sample).

The Moli-sani Study cohort was followed up until December 31st 2015 with the main outcome of interest being mortality. Overall and cause-specific mortality was assessed by the Italian mortality registry (ReNCaM registry), validated by Italian death certificates (ISTAT form) and coded according to the International Classification of Diseases (ICD-9 version).

CVD mortality included deaths from diseases of the circulatory system when the underlying cause of death included ICD9 codes 390–459. ICD-9 codes 430–438 were used to define the specific cause of death for cerebrovascular disease, ICD-9 codes 410–414 and 429 for ischemic heart disease (IHD). Cancer death was considered when the underlying cause of death included ICD9 codes 140–208. Non cardiovascular/non cancer causes of death were included in ‘other cause mortality’ group.

The Moli-sani Study complies with the Declaration of Helsinki and was approved by the ethical committee of the Catholic University Medical School in Rome, Italy. All participants provided written informed consent.

### Dietary assessment

Dietary intake was assessed by a trained interviewer-administered semi-quantitative EPIC food frequency questionnaire (FFQ) validated and adapted to the Italian population to assess participants’ diet during the past 12 months.

The FFQ includes 188 food items, classified into 74 predefined food groups on the basis of similar nutrient characteristics or culinary usage [23]. Participants were asked to indicate the number of times a given item was consumed (per day, week, month or year) from which the frequency of consumption was calculated. The quantity of food consumed was assessed by asking the participant to select one among several images of different food portions or a predefined standard portion when no image was available.

Frequencies and quantities of each food were then linked to Italian Food Tables [24], using a specifically designed software [25], to obtain quantitative estimates of daily intake of macro- and micro-nutrients plus energy.

Total egg consumption from various food sources (e.g. whole egg, omelette) was defined as number per week (we used 50 g as the standard weight for one medium-sized egg) and categorized as up to 1 egg/week, > 1 ≤ 2 eggs/week, > 2 ≤ 4 eggs/week and > 4 eggs/week or used as a continuous variable as a 1-egg increment per week.

Adherence to the traditional Mediterranean diet (MD) was evaluated by the Mediterranean Diet Score (MDS) developed by Trichopoulou et al. [26].

### Assessment of covariates

At baseline, information on socio-demographic variables, lifestyles and medical history were obtained by interviewer-administered questionnaires.

Participants were considered to have diabetes, hypertension or hyperlipidaemia at baseline if they were taking disease-specific drugs.

Leisure-time physical activity (PA) was calculated for sport activity, walking and gardening, and then dichotomized as < or ≥ 30 min/d. Bodyweight and height were measured with a column mechanical scale with a telescopic measuring rod (SECA 700, Hamburg, Germany), in subjects wearing no shoes and only light indoor clothing. Body mass index (BMI) was calculated as kg/m^2^ and then grouped into three categories as normal (≤ 25 kg/m^2^), overweight (25–30 kg/m^2^) or obese (≥ 30 kg/m^2^) [27].

Subjects were classified as never-smokers, current smokers or former smokers (quit at least 1 year ago). Education was based on the highest qualification attained and was categorized as up to lower secondary (approximately ≤ 8 years of study), upper secondary school (8–13 years of study) and postsecondary education (> 13 years of study).

Urban or rural environments were defined on the basis of the urbanization level as described by the European Institute of Statistics (EUROSTAT definition) and obtained by the tool ‘Atlante Statistico dei Comuni’ provided by the Italian National Institute of Statistics [28].

Venous blood samples were obtained from participants who had fasted overnight and had refrained from smoking for at least 6 h.

Serum lipids (total cholesterol, HDL-cholesterol, triglycerides) were assayed by enzymatic reaction methods using an automatic analyzer (ILab 350, Instrumentation Laboratory (IL), Milan, Italy).

Low-density lipoprotein (LDL) cholesterol was calculated according to Friedewald.

Quality control (high and low levels) for lipids was obtained by a commercial standard provided by the IL and an in-house serum standard pool. The coefficients of variability were respectively for high, medium and low values 4.9%, 5.2% and 4% for blood cholesterol; 3.2%, 3% and 4.5% for HDL-cholesterol; 5.2%, 5.3% and 5% for triglycerides.

### Statistical analysis

Baseline characteristics of the study population by categories of egg intake or by survival status at the end of follow-up were presented as means with standard deviation (SD), or number and percentages.

Risk estimates for all-cause and cause-specific deaths were expressed as hazard ratios (HR) with 95% confidence intervals (95% CI) and calculated using Cox regression models with time-on-study on the time scale and adjusting for baseline age as covariate in the model.

Based on previous literature and biological plausibility, two multivariable models were fitted to assess the association between egg intake and mortality: Model 1 was adjusted for age (continuous), sex and energy intake (kcal; continuous); Model 2 as in model 1 further controlled for education (up to lower secondary school; upper secondary school; postsecondary/higher), household income (≤ 10.000; > 10.000 ≤ 25.000; > 25.000 ≤ 40.000; > 40.000 EUR/y), residence (urban, rural), leisure-time PA (< 30 or ≥ 30 min/d), smoking status (never, current, former), BMI (normal, overweight, obese), diabetes (no, yes, missing), hyperlipidaemia (no, yes, missing), hypertension (no, yes, missing); Model 3 as in model 2 further controlled for MDS (continuous). Missing values for diabetes (*n* = 241), hypertension (*n* = 131) and hyperlipidaemia (*n* = 147) were included in the models as dummy variables, similar to the way valid categories were represented. For education, BMI and smoking (less than 1% of missing values) missing values were imputed to the modal value.

Main macronutrients contained in eggs (saturated fat and protein, g/d), dietary cholesterol (mg/d), dietary vitamin E (mg/d) and beta-carotene (µg/d) were tested as potential mediators of the association between egg intake and mortality.

For the mediation analysis, we used the publicly available %MEDIATE macro in SAS software [29] to calculate the point and interval estimates of the percent of exposure effect (PTE) explained by one or more intermediate variables, with 95% confidence interval and P values. Nutritional factors and serum lipids were entered into the mediation analysis as continuous variables and positively skewed variables (triglycerides) were log transformed before analysis. Mediation analyses with biomarkers were restricted to 20,146 subjects after exclusion of those individuals with missing data on any of the biomarker.

To increase the applicability of the study results we calculated risk estimations for each additional 186 mg of dietary cholesterol per day, 6.2 g of dietary proteins per day, 1.6 g of saturated fats per day, 0.56 mg of vitamin E per day and 42 µg per day of beta-carotene since these are the amounts of nutrients present in 1 egg [30].

Subgroup analyses according to levels of intake of each food group included into the MDS (above/below the study population median) and by various baseline risk factors were conducted.

Appropriate multiplicative terms for testing interaction were included in the multivariable-adjusted models to test for a difference of the effect of egg intake across subgroups.

Statistical tests were two-sided, and *p* values < 0.05 were considered to indicate statistical significance. The data analysis was generated using SAS/STAT software, version 9.4 (SAS Institute Inc., Cary, NC, USA).

## Results

### Characteristics of the population

The analysed population consisted of 10,905 women (53%) and 9657 men (47%) with an average age of 54 years (± 11 years, range 35 to 94 years). The majority of participants were low educated (51.1%), non-smokers (50.3%) and overweight (42.9%) (Table 1).

**Table 1:** Baseline characteristics of the study sample by categories of egg consumption (*n* = 20,562)

	Whole sample	Egg consumption (*n* of eggs/week)			
		> 0 ≤ 1	> 1 ≤ 2	> 2 ≤ 4	> 4
*N* of subjects (%)	20,562	5517 (26.8)	7143 (34.8)	6526 (31.7)	1376 (6.7)
Number of eggs/week, median (IQR)	1.57 (0.97–2.46)	0.63 (0.39–0.85)	1.47 (1.23–1.64)	2.55 (2.20–3.07)	4.60 (4.19–5.26)
Age, years	54 (11)	56 (11)	54 (11)	53 (11)	53 (11)
Men (%)	47.0	47.6	47.1	45.9	48.8
Educational level (%)					
Up to lower secondary	51.1	52.8	50.7	49.7	52.8
Upper secondary	35.7	33.7	36.2	36.9	35.6
Postsecondary	13.2	13.5	13.1	13.4	11.6
Income categories (%)					
≤ 10.000	5.4	6.6	5.2	4.6	5.9
> 10.000 ≤ 25.000	30.5	29.7	30.1	31.1	32.4
> 25.000 ≤ 40.000	21.1	20.7	21.5	21.0	20.5
> 40.000	12.5	12.5	13.0	12.3	10.8
Missing	30.5	30.5	30.2	31.0	30.4
Urban residence (%)	67.0	66.4	66.9	66.7	70.0
Smoking status (%)					
Non-smokers	50.3	49.9	50.4	50.6	49.7
Current smokers	23.8	22.9	23.3	24.4	27.0
Former smokers	25.9	27.2	26.2	25.0	23.3
Physical activity > 30 min/d (%)	64.0	63.8	63.5	63.8	68.3
BMI, kg/m^2^	27.9 (4.7)	28.1 (4.8)	28.0 (4.7)	27.7 (4.7)	27.7 (4.6)
BMI, kg/m^2^ (%)					
Normal (≤ 25)	28.4	27	27.1	30.4	30.7
Overweight (25–30)	42.9	42.4	43.9	42.5	41.7
Obese (≥ 30)	28.7	30.5	29.0	27.1	27.6
Diabetes (%)	4.0	4.6	4.2	3.4	3.1
Hypertension (%)	25.2	28.9	25.6	22.4	21.4
Hyperlipidaemia (%)	5.3	7.4	5.5	4.0	2.3
Total blood cholesterol, mg/dL	213.8 (40.8)	215.6 (41.8)	213.7 (40.3)	212.5 (40.3)	210.4 (40.5)
HDL-cholesterol, mg/dL	58.0 (14.7)	57.6 (14.9)	57.5 (14.6)	57.9 (14.7)	57.4 (14.7)
LDL-cholesterol, mg/dL	131.3 (34.6)	132.7 (35.5)	131.4 (34.2)	130.3 (34.4)	129.3 (34.5)
Triglycerides, mg/dL	122.4 (64.1)	126.8 (65.1)	123.9 (64.9)	121.2 (62.6)	118.4 (62.6)

1 egg = 50 g

Values are means (SDs) unless otherwise stated

Analyses for serum lipids were run on 20,146 participants

Mean consumption was 1.8 eggs per week (± 1.3), while overall mean dietary cholesterol intake was 322 mg/d (± 108). Egg contributed to 14.6% of total cholesterol intake in the diet.

Participants with a greater egg intake (> 4 eggs/week) were younger, had lower socioeconomic status, less cardiovascular risk factors (i.e. BMI, diabetes, hypertension and hyperlipidaemia), including lower levels of blood lipids than participants consuming lower amounts of egg (Table 1). High egg consumers tended to have a lower baseline MD and consumed less fruits and nuts, cereals, and more meat and meat products. The contribution of proteins and fats to total energy intake increased across categories of egg consumption, whereas that of carbohydrates and fibre decreased; dietary cholesterol increased according to egg intake, as well as vitamin E levels while no substantial differences were observed for beta-carotene levels (Supplementary Table 1).

### Egg consumption and mortality risk

The cohort of 20,562 participants was followed-up for a median of 8.3 years (interquartile range = 7.4–9.3 years; 170,032 person-years) during which 838 deaths were ascertained and validated; 271 from CVD, of which 153 were from IHD/cerebrovascular disease, 334 from cancer, and 233 from other causes (Supplementary Table 2).

In a multivariable-adjusted model including also MDS, as compared to participants reporting lower egg consumption, consuming more than 4 eggs/week was associated with increased risk of all-cause (HR = 1.50; 95%CI 1.13–1.99), CVD (HR = 1.75; 1.07–2.87) and cancer mortality (HR = 1.52; 0.99–2.33); an upward trend of risk with IHD/cerebrovascular disease mortality associated with increased egg intake was also observed (HR = 1.58; 0.79–3.17), although statistical significance was not hold (Table 2, models 3). The recommended intake of 2–4 eggs/week also led to increased all-cause (HR = 1.22; 1.01–1.46) and CVD mortality risk (HR = 1.43; 1.03–1.97) (Table 2, models 3).

**Table 2:** Hazard ratios (HR) with 95% confidence intervals (95%CI) for all-cause and cause-specific mortality associated with egg consumption in the Moli-sani Study cohort (*n* = 20,562)

	Egg consumption (*n* of eggs/week)					1 Egg/week increment	
	> 0 ≤ 1	> 1 ≤ 2	> 2 ≤ 4	> 4	*p* for trend	HR (95%CI)	*p* Value
*N* of subjects (%)	5517 (26.8)	7143 (34.8)	6526 (31.7)	1372 (6.7)		–	–
*All-cause mortality*							
*N* of deaths	234	281	256	67	–	–	–
Person-years	44,712	58,776	54,630	11,914	–	–	–
Event rates per 10,000 person-years	52.3	47.8	46.9	56.2	–	–	–
Model 1	-1-	1.13 (0.95–1.35)	1.23 (1.03–1.48)	1.55 (1.17–2.06)	0.0018	1.07 (1.01–1.13)	0.018
Model 2	-1-	1.14 (0.96–1.37)	1.21 (1.01–1.46)	1.50 (1.13–1.99)	0.0043	1.06 (1.00–1.12)	0.037
Model 3	-1-	1.14 (0.96–1.36)	1.22 (1.01–1.46)	1.50 (1.13–1.99)	0.0044	1.06 (1.00–1.12)	0.039
*CVD mortality*							
*N* of deaths	71	90	87	23	–	–	–
Event rates per 10,000 person-years	15.9	15.3	15.9	19.3	–	–	–
Model 1	-1-	1.23 (0.90–1.69)	1.43 (1.03–1.97)	1.80 (1.10–2.94)	0.0076	1.11 (1.01–1.22)	0.025
Model 2	-1-	1.29 (0.94–1.77)	1.43 (1.03–1.97)	1.75 (1.07–2.88)	0.011	1.10 (1.01–1.21)	0.036
Model 3	-1-	1.29 (0.94–1.77)	1.43 (1.03–1.97)	1.75 (1.07–2.87)	0.010	1.10 (1.01–1.21)	0.036
*IHD/ cerebrovascular mortality*							
*N* of deaths	42	51	49	11	–	–	–
Event rates per 10,000 person-years	9.4	8.7	9.0	9.2	–	–	–
Model 1	-1-	1.24 (0.82–1.87)	1.47 (0.96–2.25)	1.69 (0.85–3.38)	0.046	1.13 (1.00–1.28)	0.054
Model 2	-1-	1.26 (0.83–1.90)	1.44 (0.94–2.21)	1.58 (0.79–3.17)	0.072	1.11 (0.98–1.26)	0.090
Model 3	-1-	1.25 (0.83–1.90)	1.44 (0.94–2.21)	1.58 (0.79–3.17)	0.072	1.11 (0.98–1.26)	0.090
*Cancer mortality*							
N of deaths	94	104	106	30	–	–	–
Event rates per 10,000 person-years	21.0	17.7	19.4	25.2	–	–	–
Model 1	-1-	0.99 (0.75–1.32)	1.19 (0.89–1.58)	1.59 (1.04–2.45)	0.039	1.06 (0.97–1.15)	0.19
Model 2	-1-	0.98 (0.74–1.31)	1.16 (0.88–1.54)	1.52 (0.99–2.33)	0.070	1.05 (0.96–1.14)	0.29
Model 3	-1-	0.98 (0.74–1.30)	1.16 (0.87–1.54)	1.52 (0.99–2.33)	0.070	1.05 (0.96–1.14)	0.29
*Other causes mortality*							
N of deaths	69	87	63	14	–	–	–
Event rates per 10,000 person-years	15.8	14.2	11.4	11.8	–	–	–
Model 1	-1-	1.22 (0.89–1.68)	1.07 (0.75–1.52)	1.16 (0.64–2.11)	0.66	1.02 (0.92–1.14)	0.69
Model 2	-1-	1.24 (0.90–1.71)	1.07 (0.75–1.53)	1.16 (0.64–2.11)	0.68	1.02 (0.92–1.14)	0.71
Model 3	-1-	1.24 (0.90–1.71)	1.07 (0.75–1.53)	1.14 (0.63–2.08)	0.68	1.02 (0.92–1.13)	0.73

Model 1 adjusted for age (continuous), sex and energy intake (continuous)

Model 2 as in model 1 further adjusted for educational level (categorical), household income (categorical), residence (categorical), smoking (categorical), BMI (categorical), leisure-time PA (categorical), baseline diabetes (categorical), hypertension (categorical), hyperlipidaemia (categorical)

Model 3 as in model 2 further adjusted for the Mediterranean diet score (continuous)

**Fig. 1:**
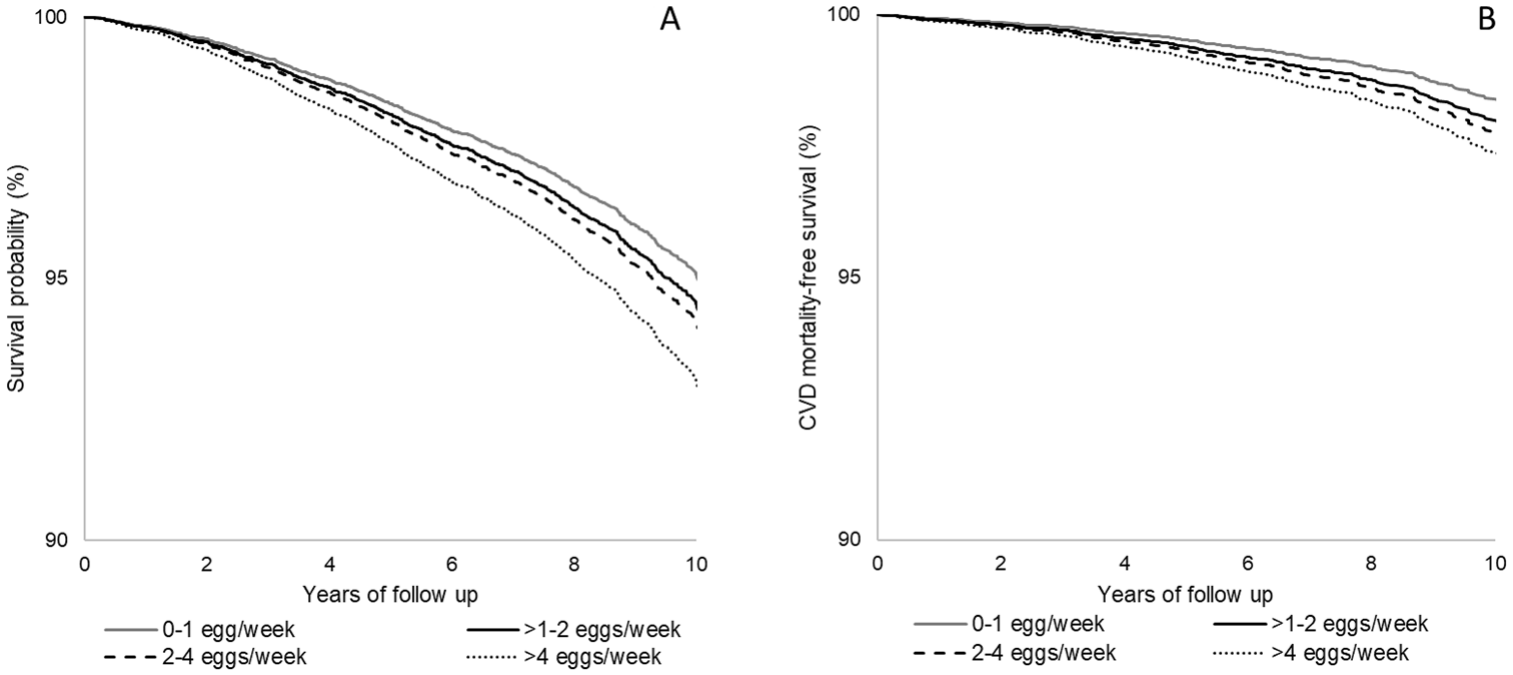
Multivariable adjusted Kaplan–Meier estimates for all-cause (**a**) and cardiovascular mortality (**b**) for increasing number of weekly egg consumption in the Moli-sani Study cohort (*n* = 20,562). Estimates were obtained from the multivariable-adjusted model including age, sex, energy intake, educational level, household income, residence, smoking, leisure-time PA, BMI, presence at baseline of diabetes, hyperlipidaemia, hypertension, and the Mediterranean diet score

Multivariable-adjusted Kaplan–Meier estimates for all-cause mortality and CVD mortality for the four categories of egg consumption are separated (Figs. 1a, b) and showed increased mortality risk associated with higher egg intake (*p* = 0.030 and *p* = 0.083 respectively).

Each additional egg per week was associated with a higher risk of all-cause (HR = 1.06; 1.00–1.12) and CVD mortality (HR = 1.10; 1.01–1.21) (Table 2, models 3).

### Dietary fats and mortality

Dietary cholesterol positively correlated with saturated fat in the diet (Spearman correlation coefficient *r* = 0.83; *p* < 0.0001, data not shown).

Each additional 186 mg of dietary cholesterol consumed per day was associated with a higher risk of all-cause (HR = 1.28; 95% CI 1.07–1.51) and CVD mortality (HR = 1.39; 95% CI 1.03–1.88; Supplementary Table, 3, model 2) although the strength of the association was reduced after inclusion of other nutrients into the model. Similarly, saturated fat intake was associated with increased risk of all-cause and CVD mortality, although adjustment for other dietary factors mitigated the magnitude of the association (Supplementary Table, 3).

### Mediation analysis

Dietary cholesterol explained 43.0% (*p* < 0.0001) and 39.3% (*p* < 0.0001) of the association of egg intake with all-cause and CVD mortality risk, respectively; saturated fats accounted from 11.6 to 13.1%, while dietary protein offered little contribution (Table 3). Antioxidant content did not attenuate the magnitude of the egg-mortality association.

**Table 3:** Nutritional factors and serum lipids as possible mediators of the association between egg intake with all-cause and cardiovascular mortality in the Moli-sani Study cohort (*n* = 20,146)

Mediator	All-cause mortality		CVD mortality	
	Proportion mediated (95% CI)	*p* value	Proportion mediated (95% CI)	*p* value
Dietary cholesterol (mg/d)	43.0% (22.8–65.7%)	< 0.0001	39.3% (16.6–67.9%)	< 0.0001
Saturated fat (g/d)	13.1% (7.7–21.4%)	< 0.0001	11.6% (5.8–21.8%)	< 0.0001
Dietary protein (g/d)	7.2% (3.8–13.0%)	< 0.0001	4.8% (1.6–13.6%)	0.016
Dietary vitamin E (mg/d)	Null	–	Null	–
Dietary beta-carotene (µg/d)	Null	–	Null	–
Dietary cholesterol, saturated fat, protein	3.0% (0.0–78.5%)	0.33	10.5% (1.3–50.4%)	0.14
Serum lipids*	6.3% (3.3–11.9%)	< 0.0001	3.8% (1.6–8.9%)	0.0009

Proportion of effect explained by intermediate variables with 95% CI and relevant *p*-value as produced by the %MEDIATE macro are reported for each potential mediator, in multivariable-adjusted Cox PH model controlled for sex, age (continuous), energy intake (continuous), educational level (categorical), household income (categorical), residence (categorical), smoking (categorical), BMI (categorical), leisure-time PA (categorical), baseline diabetes (categorical), hypertension (categorical), hyperlipidaemia (categorical), and the Mediterranean diet score (continuous)

The proportion refers to the high (> 4 eggs/week) vs low egg intake (> 0 ≤ 1 egg/week)

Null = not mediating the effect

*Mediation analysis restricted to 20,146 participants after exclusion of those individuals with missing data on any of the biomarker. Serum lipids include blood cholesterol (mg/dL), HDL-cholesterol (mg/dL), LDL-cholesterol (mg/dL), triglycerides (mg/dL; logarithm)

Dietary cholesterol inversely correlated with total serum cholesterol levels (Spearman correlation coefficients *r* = − 0.02, *p* = 0.005), HDL-cholesterol (*r* = − 0.02, *p* = 0.0005) and triglycerides (*r* = − 0.04, *p* < 0.0001) and was not related to LDL-cholesterol (*r* = 0.001, *p* = 0.92; data not shown).

Baseline differences in serum lipids across categories of egg intake marginally accounted for the association of eggs with mortality, explaining 6.3% (*p* < 0.0001) and 3.8% (*p* = 0.0009) of the relation between high egg consumption and all-cause and CVD mortality risk (Table 3).

### Sub-group analysis

Sub-group analyses indicated that overall diet quality, as measured by the MDS, and single food groups were not effect modifiers of the relation between eggs and mortality risk (all *p* values for interaction > 0.05; Supplementary Table 4), although an increased risk of CVD mortality associated with 1-egg increment per week was found among low fruit consumers as compared to high consumers (*p* for interaction = 0.0034, Supplementary Table 4).

Among baseline risk factors, hyperlipidaemia and hypertension were likely to modify the magnitude of the association between egg consumption (1-egg increment per week) and CVD mortality risk, resulting to be stronger in those using lipid-lowering drugs (*p* for interaction = 0.010) and among participants taking antihypertensive medications (*p* for interaction = 0.042; Table 4).

**Table 4:** Sub-group analysis for the association of 1 egg/week increment and all-cause and CVD mortality in the Moli-sani Study cohort (*n* = 20,562)

	All-cause mortality			CVD mortality		
	*N* of deaths/*n* of subjects	HR (95%CI)	*p *for interaction	*N* of deaths	HR (95%CI)	*p *for interaction
Whole sample	838/20,562	1.06 (1.00–1.12)	–	271	1.10 (1.01–1.20)	–
Aged < 65 years	258/16,550	1.05 (0.95–1.16)	0.37	58	1.08 (0.89–1.32)	0.44
Aged ≥ 65 years	580/4012	1.09 (1.03–1.17)		213	1.16 (1.05–1.28)	
Women	299/10,905	1.03 (0.94–1.14)	0.19	104	1.19 (1.04–1.36)	0.50
Men	539/9657	1.11 (1.04–1.18)		167	1.11 (0.98–1.24)	
Up to lower secondary education	606/10,510	1.06 (1.00–1.13)	0.88	205	1.11 (1.01–1.23)	0.64
Upper secondary/postsecondary	232/10,052	1.05 (0.94–1.16)		66	1.09 (0.88–1.34)	
Non-smokers	647/15,665	1.06 (1.00–1.13)	0.89	212	1.09 (0.99–1.21)	0.56
Smokers	191/4897	1.05 (0.94–1.17)		59	1.07 (0.88–1.29)	
Physical activity ≤ 30 min/d	306/7402	1.07 (0.97–1.17)	0.49	101	1.10 (0.94–1.28)	0.81
Physical activity > 30 min/d	532/13,160	1.06 (0.99–1.13)		170	1.11 (0.99–1.24)	
Normal/overweight	549/14,655	1.07 (1.00–1.14)	0.81	165	1.12 (1.00–1.26)	0.69
Obese	289/5907	1.07 (0.98–1.17)		106	1.09 (0.94–1.26)	
Free from diabetes	701/19,501	1.06 (1.00–1.12)	0.82	224	1.09 (0.99–1.20)	0.20
Subjects with diabetes	113/820	1.09 (0.93–1.28)		34	1.26 (0.95–1.65)	
Free from hyperlipidaemia	762/19,323	1.06 (1.00–1.12)	0.97	250	1.07 (0.97–1.18)	0.010
Subjects with hyperlipidaemia	66/1092	1.06 (0.86–1.32)		18	1.66 (1.21–2.26)	
Free from hypertension	435/15,256	1.03 (0.96–1.11)	0.35	107	1.00 (0.85–1.18)	0.042
Subjects with hypertension	400/5175	1.08 (1.00–1.17)		163	1.15 (1.03–1.28)	
Excluding early deaths (follow up > 2 years)	740/20,464	1.06 (1.00–1.12)	–	233	1.14 (1.01–1.22)	–

Hazard ratios with 95% CI from the multivariable model adjusted for sex, age (continuous), energy intake (continuous), educational level (categorical), household income (categorical), residence (categorical), smoking (categorical), BMI (categorical), leisure-time PA (categorical), baseline diabetes (categorical), hypertension (categorical), hyperlipidaemia (categorical), and the Mediterranean diet score (continuous)

Missing data: diabetes (*n* = 241), hypertension (*n* = 131) and hyperlipidaemia (*n* = 147)

## Discussion

In a large cohort of Italian adults, eating more than 4 eggs per week was associated with an increased risk of all-cause and CVD mortality, in comparison with a lower intake (up to 1 egg per week) and independently of overall diet quality as reflected by adherence to the Mediterranean diet. Of interest, we found that even moderate egg intake of 2–4 servings per week, which is generally recommended by international dietary guidelines, led to an increased risk of all-cause and CVD mortality by 22% and 43%, respectively.

Egg and dietary cholesterol intakes in our cohort were similar to that reported in European countries from EPIC study [31] and in some US cohorts [32, 33], but egg consumption in our cohort was slightly lower than that documented in other Mediterranean cohorts, which reported an average intake of 3–4 eggs per week [9, 19].

Our findings expand the scarce literature on this topic in Mediterranean countries. Consumption of 10 g of egg per day (corresponding to approximately 1 and a half egg per week) was found associated with increased risk of total (31%) and CVD death (54%) in patients with diabetes from the Greek arm of the EPIC study [21], while in the Spanish cohort of EPIC no association between egg consumption (up to 7 eggs/week) and all-cause, CVD and IHD of mortality was found; yet, inverse associations between egg intake and risk of death from other causes (24% reduction) and from the nervous system diseases (41%) were documented [20]. In the Spanish SUN cohort, no association was found between egg consumption and the incidence of CVD [9].

Studies from non-Mediterranean cohorts also yielded inconsistent findings. In a recent analysis from the NHANES cohort no significant associations between egg consumption and mortality in US adults were observed [34]; on the contrary, pooled data from 6 US-based cohorts showed that each additional half an egg consumed per day was significantly associated with 6% higher risk of incident CVD and 8% of all-cause mortality [32].

Moreover, recent findings from nine European countries showed that higher egg consumption was associated with a higher risk of haemorrhagic stroke (25%) and an upward trend of increased ischaemic stroke risk [31], and in a Japanese cohort of women, a direct association was reported between egg intake (≥ 2 eggs/d vs 1 egg/d) and total mortality [17].

A meta-analysis of 14 studies involving 320,778 subjects identified a dose–response positive association between egg consumption and the risk of CVD and diabetes [12], but more recently an analysis from three large US cohorts and an updated meta-analysis including 28 prospective studies showed that moderate egg consumption (up to 7 eggs per week) was not associated with cardiovascular disease risk overall [35].

Recently, a dose–response meta-analysis of prospective cohort studies found no association with risk of cardiovascular outcomes following the habitual consumption of one egg per day compared to no intake, with exception of the risk of heart failure, which resulted higher especially in men from US cohorts [36].

Inconsistencies across studies may be due to differences in population characteristics, sample sizes, cooking methods for eggs, or differences in dietary patterns related to different amounts of egg consumption and also different adjustments for confounders.

Our study relied on a comprehensive assessment of nutritional factors (e.g. saturated fats and overall diet quality) and also on a number of other potential confounding variables, such as lifestyles and socio-demographic factors.

We also observed an increased risk of cancer mortality associated with eating more than 4 eggs per week and this is in line with a dose–response meta-analysis of prospective observational studies showing that eating ≥ 5 eggs per week may be associated with a modestly elevated risk of breast, ovarian and fatal prostate cancers [37]. Similarly, Japanese women consuming more than 2 eggs per day, as compared to those having 1 egg/d, tripled their risk of dying from cancer [17].

Furthermore, we found that the association between egg consumption and CVD mortality looked much stronger among subjects with hyperlipidaemia, suggesting that egg consumption should be strongly discouraged in this high-risk subgroup, even despite the use of lipid-lowering medications.

Generally, eggs are a controversial food because of their saturated fat (about 3 g/100 g) and cholesterol content (about 370 mg/100 g) [30] and on this basis experts have produced mounting evidence against frequent egg consumption due to their potential association with CVD [32].

Indeed, pooled data from 6 US-based cohorts recently showed that each additional 300 mg of dietary cholesterol consumed per day was significantly associated with 17% and 18% higher risk of incident CVD and all-cause mortality, respectively [32], while others found that the dietary cholesterol-mortality relation likely depends on the baseline intake levels, with an inverse association in those with lower intake levels (< 250 mg/day) but a positive association in those with higher intake levels (≥ 250 mg/day) [34].

In our study, we found that a substantial part of the excess risk of all-cause and CVD mortality associated with egg intake was accounted for by dietary cholesterol that, in turn, was associated with an increased risk of all-cause and CVD mortality, although the strength of the relation was reduced in multivariable-adjusted models. Our data are in accordance with observational data from US cohorts reporting that dietary cholesterol largely explained the association of eggs with increased CVD mortality [32].

However, the potential health risk of high dietary cholesterol levels has been questioned [38, 39] and more recently a meta-analysis concluded that available evidence is too heterogeneous and actually lacks methodologic rigor to draw any definitive conclusion regarding the influence of dietary cholesterol on CVD risk [38].

Since cholesterol-containing foods are usually rich in saturated fat and animal protein, which have been associated with increased CVD mortality risk in previous reports [40, 41], we also accounted for such nutrients but found that proteins were unlikely to attenuate the relationship between eggs and mortality risk, while saturated fats played a limited role. These results should be interpreted in light of the fact that eggs contain high-quality protein with minimal saturated fatty acids.

Differences at baseline in serum lipids among study participants were unlikely to explain the excess of CVD risk associated with higher egg intake while explaining about 7% of the relation with all-cause mortality. Individuals eating eggs more frequently tended to have lower levels of serum lipids; this may be counter-intuitive, but epidemiological evidence on a direct association between serum lipids and disease/death risk is inconsistent and not fully elucidated [42, 43].

### Strengths and limitations

To our knowledge, this is one of the largest prospective cohort studies evaluating the association between egg consumption and mortality in a Mediterranean population, and one of the few examining the role of the nutrient content of eggs in the egg- mortality relation.

Major strengths of this study include a large community-based cohort, its prospective design, detailed information of dietary intake and the considerable number of covariates allowing to minimize sources of bias and confounding.

However, this study also suffers from several limitations: first, the observational nature of the study cannot allow to fully rule out residual confounding or confounding by unmeasured factors. Second, cause-specific mortality analysis in this dataset is limited by the small number of deaths and the relatively short period of follow up; also, the rather small number of subjects and events in the highest egg consumption category has to be acknowledged.

Furthermore, information on dietary intake was self-reported and can lead to under- or over-estimates; moreover, subjects’ dietary information was collected at baseline only, thus life-course changes possibly occurred during the follow-up period, may have influenced the strength of our findings. Also, we do not have data on preferred cooking methods which may likely influence the egg-mortality association.

Finally, participants lived in Molise, a region located between central and southern Italy, that its traditionally Mediterranean in culture; thus caution is needed in extending our results to other geographical and cultural contexts, although the main characteristics of our population sample are comparable to those of the Italian Cardiovascular Epidemiological Observatory and therefore representative of at least the Italian population [44].

## Conclusions

Our findings report an increased risk of all-cause, CVD and cancer mortality associated with intake of more than 4 eggs per week in a large cohort of Italians from the general population. Increased death risk was also observed at lower intakes, namely 2–4 servings per week, which correspond to the egg intake recommended by many health bodies and international dietary guidelines.

The adverse health effects of eggs were found to be independent of the overall quality of the diet, while part of the excess of all-cause and CVD mortality risk was likely due to the high content of cholesterol and saturated fats of eggs, which in turn were found predictive of higher mortality risk in our cohort. As dietary cholesterol was not directly related to serum lipids, its adverse health effect may likely pass through biological mechanisms other than cholesterol-related ones.

Although we do acknowledge the limitations of our observational study, our findings are unlikely to be supportive of the current dietary guidelines most of which recommending a safe use up to 4 eggs per week, or even no type of restriction.

Finally, caution should be taken especially for high-risk individuals, such as those with hypercholesterolemia and hypertension.

## Supplementary Information

Below is the link to the electronic supplementary material.

Supplementary file1 (DOC 141 KB)
