# Supplementary material for: Egg consumption and risk of all-cause and cause-specific mortality in an Italian adult population
Source: Eur J Nutr. 2021 Mar 24;60(7):3691–702. doi: 10.1007/s00394-021-02536-w (PMC8437843; doi:10.1007/s00394-021-02536-w)
Supplement: Supplementary file 1 — Supplementary file1 (DOC 141 KB) [file 394_2021_2536_MOESM1_ESM.doc]

**Supplementary Table 1.** Dietary factors across categories of egg consumption in the Moli-sani Study cohort (n=20,562)

|  | **Egg intake (n of eggs/week)** | | | | |
| --- | --- | --- | --- | --- | --- |
|  | >0 ≤1 | >1 ≤2 | >2 ≤4 | >4 | P value |
| N of subjects ( %) | 5517 (26.8) | 7143 (34.8) | 6526 (31.7) | 1376 (6.7) | - |
| Mediterranean diet score | 4.42 (1.64) | 4.37 (1.65) | 4.33 (1.63) | 4.36 (1.64) | 0.023 |
| *Food groups (g/d)* |  |  |  |  |  |
| Vegetables | 160.3 (71.7) | 160.7 (70.7) | 157.9 (68.4) | 164.2 (78.5) | 0.0051 |
| Fruits and nuts | 359.4 (202.0) | 355.1 (197.2) | 351.8 (200.6) | 335.2 (205.0) | 0.0005 |
| Legumes | 26.9 (20.6) | 27.0 (20.9) | 27.0 (20.0) | 29.0 (21.4) | 0.0033 |
| Cereals | 216.8 (89.8) | 211.0 (92.3) | 201.0 (90.3) | 187.5 (98.1) | <.0001 |
| Fish | 44.0 (27.9) | 44.0 (25.0) | 45.4 (25.6) | 46.0 (27.6) | 0.0009 |
| Meat and meat products | 99.3 (41.8) | 104.4 (42.8) | 107.7 (43.1) | 111.6 (52.4) | <.0001 |
| Milk and dairy products | 182.7 (122.9) | 187.2 (121.0) | 185.2 (123.1) | 183.7 (128.2) | 0.17 |
| Monounsaturated/saturated fatty acids | 1.41 (0.33) | 1.38 (0.28) | 1.37 (0.25) | 1.40 (0.26) | <.0001 |
| Moderate alcohol intake (%) | 34.1 | 37.4 | 37.9 | 38.7 | <.0001 |
| Energy intake (Kcal/d) | 1917 (541) | 2093 (551) | 2223 (557) | 2440 (585) | <.0001 |
| *Macronutrients and nutrients* |  |  |  |  |  |
| Carbohydrate (% energy intake) | 50.2 (7.5) | 48.7 (6.7) | 47.4 (6.5) | 45.9 (6.4) | <.0001 |
| Protein (% energy intake) | 15.8 (2.3) | 16.2 (2.1) | 16.4 (2.1) | 16.8 (2.0) | <.0001 |
| Fat (% energy intake) | 32.0 (6.0) | 32.9 (5.5) | 33.9 (5.4) | 35.4 (5.4) | <.0001 |
| Saturated fats (% energy intake) | 11.4 (2.8) | 11.8 (2.5) | 12.2 (2.5) | 12.5 (2.6) | <.0001 |
| Monounsaturated fats (% energy intake) | 15.5 (3.2) | 15.8 (2.9) | 16.3 (2.8) | 17.0 (2.8) | <.0001 |
| Polyunsaturated fats (% energy intake) | 3.4 (0.7) | 3.5 (0.6) | 3.6 (0.6) | 3.8 (0.6) | <.0001 |
| Fiber (g/d) | 20.8 (6.5) | 20.4 (6.5) | 20.0 (6.5) | 19.7 (6.7) | <.0001 |
| Dietary cholesterol (mg/d) | 269.7 (84.6) | 306.8 (82.5) | 355.4 (86.2) | 444.9 (105.2) | <.0001 |
| Vitamin E (mg/d) | 7.0 (2.1) | 7.0 (2.1) | 7.1 (2.1) | 7.3 (2.2) | <.0001 |
| Beta-carotene (µ/d) | 2671 (1350) | 2682 (1332) | 2652 (1299) | 2770 (1534) | 0.018 |

1 egg = 50 g.

Values are means (SDs) unless otherwise stated.

Means and P values adjusted for sex, age (continuous) and energy intake (continuous).

**Supplementary Table 2.** Baseline characteristics categorized by survival status at the end of follow-up in the Moli-sani Study cohort (n=20,562)

|  | Alive | Dead (all-cause) | Dead from CVD | Dead from cancer |
| --- | --- | --- | --- | --- |
|  |  |  |  |  |
| Mean number of eggs/week (min-max) | 1.8 (0.0-17.8) | 1.8 (0.0-9.9) | 1.9 (0.0-9.9) | 1.8 (0.0-6.1) |
| N of subjects (%) | 19,726 | 838 | 271 | 334 |
| Age, years | 54 (11) | 70 (11) | 71 (11) | 65 (10) |
| Men (%) | 46.2 | 64.3 | 61.6 | 66.8 |
| Educational level (%) |  |  |  |  |
| *Up to lower secondary* | 50.2 | 72.3 | 75.6 | 68.6 |
| *Upper secondary* | 36.4 | 20.2 | 15.5 | 23.6 |
| *Postsecondary* | 13.4 | 7.5 | 8.9 | 7.8 |
| Income categories (%) |  |  |  |  |
| *≤10.000* | 5.2 | 12.2 | 14.4 | 8.7 |
| >*10.000≤25.000* | 30.4 | 31.7 | 29.1 | 30.2 |
| *>25.000≤40.000* | 21.5 | 11.1 | 10.3 | 12.6 |
| *>40.000* | 12.7 | 7.0 | 5.2 | 9.6 |
| *Missing* | 30.2 | 38.0 | 41.0 | 38.9 |
| Urban residence (%) | 66.7 | 71.2 | 72.3 | 71.0 |
| Smoking status (%) |  |  |  |  |
| *Non-smokers* | 50.7 | 41.0 | 41.7 | 38.0 |
| *Current smokers* | 23.9 | 22.8 | 21.8 | 27.6 |
| *Former smokers* | 25.4 | 36.2 | 36.5 | 34.4 |
| Physical activity >30 min/d (%) | 64.0 | 63.5 | 62.7 | 66.8 |
| BMI, kg/m^2^ (%) |  |  |  |  |
| *Normal (≤25)* | 28.6 | 22.7 | 20.7 | 21.9 |
| *Overweight (25-30)* | 42.9 | 42.8 | 40.2 | 43.7 |
| *Obese (≥30)* | 28.5 | 35.5 | 39.1 | 34.4 |
| Diabetes (%) | 3.6 | 13.5 | 12.6 | 14.4 |
| Hypertension (%) | 24.2 | 47.7 | 60.2 | 39.8 |
| Hyperlipidaemia (%) | 5.2 | 7.9 | 6.6 | 9.3 |
| Total blood cholesterol, mg/dL | 214.2 (40.7) | 205.1 (41.9) | 206.4 (41.4) | 208.8 (39.2) |
| HDL-cholesterol, mg/dL | 58.1 (14.7) | 56.1 (15.0) | 56.1 (15.1) | 55.9 (14.3) |
| LDL-cholesterol, mg/dL | 131.7 (34.6) | 123.7 (35.0) | 125.0 (34.5) | 127.5 (34.2) |
| Triglycerides, mg/dL | 122.2 (64.2) | 126.6 (63.0) | 126.5 (62.6) | 126.8 (60.7) |

1 egg = 50 g.

Values are means (SDs) unless otherwise stated.

Means are adjusted for sex, age (continuous) and energy intake (continuous).

Analyses for serum lipids were run on 20,146 participants.

**Supplementary Table 3.** Hazard ratios (HR) with 95% confidence intervals (95%CI) for all-cause and cardiovascular (CVD) mortality associated with dietary cholesterol (186 mg/d increment) and saturated fat (1.6 g/d increment) intake in the Moli-sani Study cohort (n=20,562)

|  | **Dietary cholesterol (mg/d)** | | **Saturated fat (g/d)** | |
| --- | --- | --- | --- | --- |
|  | All-cause mortality | CVD mortality | All-cause mortality | CVD mortality |
|  |  |  |  |  |
| Model 1 (HR, 95%CI) | 1.294 (1.090-1.536) | 1.440 (1.065-1.947) | 1.022 (1.002-1.043) | 1.029 (0.994-1.067) |
| Model 2 (HR, 95%CI) | 1.275 (1.073-1.514) | 1.391 (1.029-1.880) | 1.025 (1.005-1.045) | 1.029 (0.993-1.066) |
| **Model 2 + nutrients (HR, 95%CI)** |  |  |  |  |
| Dietary cholesterol (mg/d) | - | - | 1.012 (0.987-1.038) | 1.009 (0.965-1.055) |
| SFAs (g/d) | 1.196 (0.963-1.487) | 1.331 (0.913-1.939) | - | - |
| MUFAs (g/d) | 1.339 (1.110-1.616) | 1.462 (1.055-2.026) | 1.038 (1.013-1.063) | 1.041 (0.998-1.087) |
| PUFAs (g/d) | 1.387 (1.145-1.681) | 1.518 (1.086-2.120) | 1.034 (1.011-1.057) | 1.037 (0.998-1.078) |
| Protein (g/d) | 1.258 (1.022-1.548) | 1.462 (1.017-2.103) | 1.022 (0.998-1.046) | 1.030 (0.987-1.076) |
| Fibre (g/d) | 1.271 (1.064-1.517) | 1.433 (1.051-1.956) | 1.024 (1.004-1.045) | 1.033 (0.995-1.072) |
| Sodium (mg/d) | 1.260 (1.060-1.498) | 1.397 (1.031-1.892) | 1.023 (1.003-1.044) | 1.030 (0.993-1.068) |
| All nutrients | 1.307 (1.024-1.668) | 1.544 (1.015-2.348) | 1.024 (0.993-1.055) | 1.032 (0.978-1.089) |
| Mediterranean diet score | 1.227 (1.029-1.462) | 1.383 (1.017-1.880) | 1.017 (0.996-1.039) | 1.029 (0.990-1.069) |

Model 1 adjusted for age (continuous), sex and energy intake (continuous).

Model 2 as in model 1 further adjusted for educational level (categorical), household income (categorical), residence (categorical), smoking (categorical), BMI (categorical), leisure-time PA (categorical), baseline diabetes (categorical), hypertension (categorical), hyperlipidaemia (categorical).

**Supplementary Table 4.** Association of 1 egg/week increment with all-cause and cardiovascular (CVD) mortality across food group intakes in the Moli-sani Study cohort (n=20,562)

|  | **All-cause mortality** | | **CVD mortality** | |
| --- | --- | --- | --- | --- |
|  | N of deaths/  n of subjects | HR (95%CI) | N of deaths | HR (95%CI) |
| Whole study sample | 838/2,0562 | 1.06 (1.00-1.12) | 271 | 1.10 (1.01-1.20) |
| Low consumption of vegetables | 503/10,328 | 1.03 (0.95-1.11) | 176 | 1.15 (1.03-1.30) |
| High consumption of vegetables | 335/10,234 | 1.09 (1.01-1.17) | 95 | 1.05 (0.91-1.21) |
| *P for interaction* |  | *0.26* |  | *0.47* |
| Low consumption of fruits | 427/10,327 | 1.09 (1.01-1.17) | 133 | 1.26 (1.12-1.42) |
| High consumption of fruits | 411/10,235 | 1.03 (0.96-1.12) | 138 | 0.96 (0.84-1.10) |
| *P for interaction* |  | *0.45* |  | *0.0034* |
| Low consumption of cereals | 515/10,489 | 1.07 (1.00-1.16) | 175 | 1.16 (1.03-1.31) |
| High consumption of cereals | 323/10,073 | 1.04 (0.96-1.13) | 96 | 1.01 (0.87-1.17) |
| *P for interaction* |  | *0.86* |  | *0.31* |
| Low consumption of legumes | 384/10,353 | 1.06 (0.97-1.15) | 107 | 1.05 (0.89-1.23) |
| High consumption of legumes | 454/10,209 | 1.06 (0.99-1.14) | 164 | 1.14 (1.03-1.27) |
| *P for interaction* |  | *0.79* |  | *0.30* |
| Low consumption of meat | 310/10,210 | 1.10 (1.02-1.19) | 89 | 1.15 (1.00-1.32) |
| High consumption of meat | 528/10,352 | 1.03 (0.95-1.10) | 182 | 1.06 (0.94-1.19) |
| *P for interaction* |  | *0.58* |  | *0.67* |
| Low consumption of fish | 461/10,286 | 1.07 (1.00-1.15) | 152 | 1.08 (0.96-1.22) |
| High consumption of fish | 377/10,276 | 1.05 (0.97-1.14) | 119 | 1.12 (0.98-1.28) |
| *P for interaction* |  | *0.61* |  | *0.64* |
| Low consumption of dairy | 409/10,183 | 1.09 (1.01-1.17) | 127 | 1.14 (1.01-1.28) |
| High consumption of dairy | 429/10,379 | 1.02 (0.94-1.11) | 144 | 1.07 (0.93-1.22) |
| *P for interaction* |  | *0.17* |  | *0.30* |
| Low ratio monounsaturated/  saturated fat | 395/10,050 | 1.05 (0.97-1.14) | 133 | 1.07 (0.94-1.22) |
| High ratio monounsaturated/  saturated fat | 443/10,512 | 1.06 (0.99-1.15) | 138 | 1.13 (1.00-1.29) |
| *P for interaction* |  | *0.66* |  | *0.67* |
|  |  |  |  |  |
| Low consumption of alcohol | 482/13,003 | 1.05 (0.97-1.13) | 143 | 1.15 (1.01-1.31) |
| High consumption of alcohol | 356/7,559 | 1.07 (0.99-1.16) | 128 | 1.06 (0.93-1.21) |
| *P for interaction* |  | *0.50* |  | *0.41* |
| Poor MDS (0-4) | 445/10,947 | 1.08 (1.00-1.16) | 143 | 1.15 (1.01-1.30) |
| Good MDS (5-9) | 393/9,615 | 1.04 (0.97-1.12) | 138 | 1.06 (0.93-1.21) |
| *P for interaction* |  | *0.74* |  | *0.29* |

Hazard ratio (HR) with 95% confidence interval for high (>4 eggs/week) vs low egg consumers (>0 ≤1 egg/week) from the multivariable-adjusted controlled for sex, age (continuous), energy intake (continuous), educational level (categorical), household income (categorical), residence (categorical), smoking (categorical), BMI (categorical), leisure-time PA (categorical), baseline diabetes (categorical), hypertension (categorical), hyperlipidaemia (categorical) and the Mediterranean diet score (alternately deprived of each food group).
High and low consumption of food groups: below or above the sex specific median of the sample.
